# Visualising disease progression on multiple variables with vector plots and path plots

**DOI:** 10.1186/1471-2288-9-32

**Published:** 2009-05-27

**Authors:** Stanley E Lazic, Sarah L Mason, Andrew W Michell, Roger A Barker

**Affiliations:** 1 Cambridge Computational Biology Institute, Department of Applied Mathematics and Theoretical Physics, University of Cambridge, CB3 0WA, UK; 2 Centre for Brain Repair, University of Cambridge, CB2 0PY, UK; 3 Department of Clinical Neurophysiology, National Hospital for Neurology and Neurosurgery, Queen Square, London, WC1N 3BG, UK; 4 Department of Neurology, Addenbrooke's Hospital, Cambridge CB2 0QQ, UK

## Abstract

**Background:**

It is often desirable to observe how a disease progresses over time in individual patients, rather than graphing group averages; and since multiple outcomes are typically recorded on each patient, it would be advantageous to visualise disease progression on multiple variables simultaneously.

**Methods:**

A variety of vector plots and a path plot have been developed for this purpose, and data from a longitudinal Huntington's disease study are used to illustrate the utility of these graphical methods for exploratory data analysis.

**Results:**

Initial and final values for three outcome variables can be easily visualised per patient, along with the change in these variables over time. In addition to the disease trajectory, the path individual patients take from initial to final observation can be traced. Categorical variables can be coded with different types of vectors or paths (e.g. different colours, line types, line thickness) and separate panels can be used to include further categorical or continuous variables, allowing clear visualisation of further information for each individual. In addition, summary statistics such as mean vectors, bivariate interquartile ranges and convex polygons can be included to assist in interpreting trajectories, comparing groups, and detecting multivariate outliers.

**Conclusion:**

Vector and path plots are useful graphical methods for exploratory data analysis when individual-level information on multiple variables over time is desired, and they have several advantages over plotting each variable separately.

## Background

Clinical studies typically measure multiple outcomes on patients as well as record information on patient characteristics such as age, sex, genotype, disease severity, and age of onset. Many such studies are longitudinal, where initial or baseline values are obtained, and then patients are followed over time to observe how the disease progresses. Often the research question involves a comparison of two or more groups, such as an experimental and control group, or a comparison of progression between subgroups of patients with the disease. Numerous methods are available to analyse multiple observations on subjects over time, such as repeated measures ANOVA, multivariate ANOVA, derived-variable or summary-measure analysis (e.g. slopes, intercepts, area under the curve, etc. [1]), time-series analysis, mixed-effects models [2], and functional data analysis [3,4], with the data often being graphically presented as either a line or bar graph, where the mean (averaged across subjects) and standard error of the mean are plotted at each time point. Alternatively, separate lines for each patient are occasionally used to show how individual patients change over time.

There is however a comparative lack of graphical methods to visualise more than one variable at multiple time points; this would be useful to help understand how individual patients progress on two or three variables simultaneously, and how each individual compares to the mean of their respective group or to all other patients. Current multivariate methods – both supervised and unsupervised – and associated graphical techniques mainly focus on finding groups, classes, clusters, or structure in the data, but generally do not consider changes over time on these variables [5,6]. If time is included as a variable then 'multivariate' generally refers to multiple observations on a *single variable*, and the ability to visualise *multiple observations *on *multiple variables *for each individual would be of great use in understanding the results of many biomedical studies. This would be useful for exploratory data analysis (EDA), as it would allow for the detection of bivariate or multivariate outliers – patients whose values on any single variable are within the normal range, but whose values on a combination of variables is unusual. For example, a value of 189 cm (6'2") is well within the normal adult range for height, as is 59 kg (130 lbs) for weight; however, it would be unusual for *the same person *to have a height of 189 cm and a weight of only 59 kg. In practice, height and weight are combined and expressed as a body mass index (BMI = kg/m^2^), and this individual's very low BMI could be detected with standard methods. However, most combinations of variables do not have such conventions to relate them to each other, and they cannot be easily expressed by a convenient method such as a product or sum. For example, in a patient with Huntington's disease (HD), there is no meaningful way to combine performance on a cognitive test such as the cognitive score of the Unified Huntington's Disease Rating Scale (UHDRS) with dopamine D_2 _receptor density in the striatum, as determined by PET imaging using ^11^C raclopride [7-9]. Multivariate outlier detection is a necessary quality control step prior to statistical analysis, as it draws attention to data that may have been recorded incorrectly and which would not be detected by examining the values for each variable separately using standard graphical methods such as histograms or quantile plots. In addition, EDA allows for the detection of novel or interesting relationships in the data – relationships that may not have been predicted beforehand, and which might go unnoticed with standard analytical methods.

Due to the work of Tukey [10], Cleveland [11,12], Cook and Swayne [13], and many others [14], it is now widely recognised that to fully appreciate the structure of data it must be examined visually. This is particularly true of multivariate data, and therefore we have developed several variations of a standard vector plot and used these to visualise disease progression in a cohort of patients with Huntington's disease from a recent paper by Michell et al. [15], especially the data shown in Figure three of that paper.

Vector plots are often used to graph information on wind speed and direction, fluid flow, magnetic fields, or to examine the behaviour of systems of differential equations. Typically, the base of the vectors (arrows) are arranged on a grid, and the length and/or thickness of the vectors encodes information on magnitude (e.g. wind speed), while the direction of the vectors relates to the direction of the phenomenon. In the neuroscience literature, vector plots have been used to represent the response of neurons in the motor cortex to movement in a particular direction, with the length of the vectors corresponding to the firing rate of the neurons [[16], p. 390–391]. In our graphs, the base of the arrows are not arranged on a grid but encode information on the initial values of multiple variables and the tips of the arrows are the final values on these same variables, with one vector for each patient. The length of the arrows therefore encodes the magnitude of change over time from initial to final values, while the direction of the vectors indicates the direction of change (increasing/decreasing, better/worse, etc. depending on what is being graphed). Since each vector represents one individual, it is possible to view how individuals change over time as well as how each individual compares to the rest of the sample. The length and direction of the vectors can be thought of as a disease trajectory, showing how patients progress in 'disease space' on multiple variables (assuming that these variables suitably reflect the disease state). Six pieces of information per patient can be easily visualised: initial and final values for three variables on a 3D graph, and further variables such as group membership (e.g. male vs. female) can be encoded by different types of vectors, for example vectors of different colour. Separate panels can also be used to plot additional categorical or continuous variables. The vector plots graphically display the net change from initial to final observation, but do not provide information on the route or path taken between these two time points. A 'path plot' is therefore introduced which is similar in principle to a vector plot, but it traces the progression of the disease over time.

The usefulness of examining patient-level data is recognised [17], and is particularly suitable for the evaluation of biomarkers, as it is not enough that a marker reliably tracks the progression of the disease on average, but that it does so sufficiently well for each individual patient [18]. In addition, it is likely that combinations of biomarkers may prove to be a more powerful method for following disease progression.

## Methods

### Patients and apparatus

Huntington's disease is a progressive neurodegenerative disorder caused by an increase in the number of glutamine amino acids in the huntingtin protein, due to an expansion in the number of CAG repeats encoding for glutamine in the first exon of the *huntingtin *gene. HD affects approximately 1 in 10,000 people with onset typically occurring in the fourth decade and progresses for some 10–20 years before becoming fatal [19]. Patients with genetically confirmed disease were recruited from the regional HD clinic at the Cambridge Centre for Brain Repair and were assessed every six months on the Unified Huntington's Disease Rating Scale (UHDRS [20]), Total Functional Assessment scale (TFA) and a hand tapping task; further information can be found in Michell et al. [15,21]. Approval was obtained from the ethical review committee at Addenbrooke's Hospital, Cambridge, (Reference number: LREC95/086) in compliance with the Helsinki Declaration, and informed consent was obtained from the patients.

The tapping apparatus consisted of two buttons 6 cm in diameter, mounted with their centres 30 cm apart. The patients' task was to alternately tap one button after the other as rapidly as possible using the palm of one hand. The total number of taps made in 30 seconds was recorded for each hand, and the data are presented as the mean of the left hand and right hand scores. The UHDRS is a uniform assessment of the clinical features of HD and contains a motor function subscale, which measures patients' ability on a range of motor tasks including eye movements, speech, tongue protrusion, bradykinesia, dystonia, chorea, and gait. Asymptomatic individuals and controls have a value of zero and higher values indicate worse performance, with a maximum score of 120 on the motor subscale. The TFA is series of twenty five questions which assesses patients' functioning on five areas including work, finances, domestic chores, activities of daily living, and the level of care required. The scores range from 0–50, with higher scores indicating worse performance.

### Graphics and analysis

Figures were created with R (version 2.8.0) [22,23], with code for the bivariate IQR ellipses adapted from Everitt [6]. The initial and final scores are provided in Additional File 1 and longitudinal data are provided in Additional File 2. R functions for some of the graphs are provided in Additional File 3 and information on the R language can be found in Venables and Ripley [24] or Crawley [25] as well as on the R website http://www.r-project.org. A good discussion of R graphing commands can be found in Murrell [26].

## Results and Discussion

### Visualising raw data

**Figure 1:**
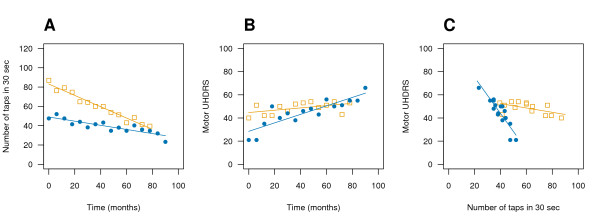
**Longitudinal tapping and UHDRS data for two individuals**. There is a steady decline in the number of taps that these two patients are able to perform, with the orange patient (squares) decreasing at a faster rate (A). UHDRS scores gradually increase (worsen) over time, but it is the blue patient (circles) that is deteriorating faster on this measure (B). There is also a good correlation between the two outcomes (C), but this plot loses any reference to time and how these variables represent disease progression. It would be difficult to examine many patients simultaneously on these two measures over time in order to observe trends. These same two individuals are also highlighted (by colour) in Figures 2A, 3A and 3B so that it can be seen how this raw data is represented by vectors.

**Figure 2:**
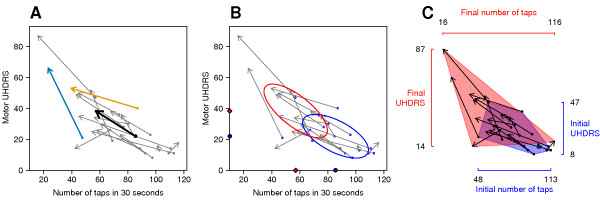
**Vector plots of raw data with various summary statistics**. The base of the arrows (closed circles) are the number of taps (*x*-axis) and UHDRS scores (*y*-axis) upon entry into the study (A). The tip of the arrows represent the number of taps and UHDRS scores several years later. For example, the orange patient could perform 85 taps initially, but only 40 at the final assessment (compare with Fig 1A), whereas this patient had an initial UHDRS score of approximately 40 and a final score of 50 (compare with Fig 1B). The length of the arrow has a straightforward interpretation: the longer the arrow the greater the disease progression, and with this dataset arrows pointing up and to the left indicate disease progression. The black arrow is the mean vector, and thus offers a visual summary of the raw data. In graph (B) the initial values are highlighted by plotting them in a different colour, and instead of a mean vector, the mean of the initial (blue circles) and final (red diamonds) values are shown on the axes. The ellipses are the 2D interquartile range of the initial (blue) and final (red) values. In graph (C) the axes show the range of the data for both initial and final values and convex polygons enclose the initial (blue) and final (red) values, which allows easier visual separation of the data at both time points.

**Figure 3:**
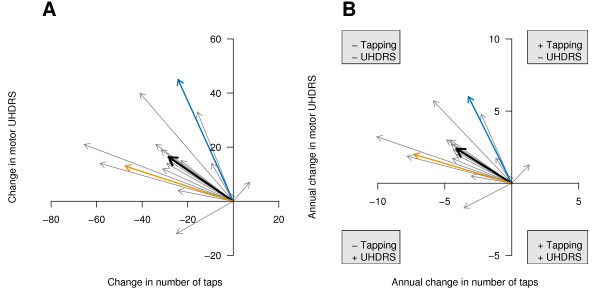
**Vector plots of change scores**. Data are changed from initial values (A) and normalised for different lengths of follow-up time and shown as the average change per year (B). The black arrow is the mean vector in both graphs and the length of the arrows in (B) are directly comparable. It can be seen that some patients progress much faster than others. The top left quadrant represents disease progression on both the tapping and UHDRS scores, with 15 of the 17 patients progressing on both of these measures. Patients that do not follow the general trend stand out: one patient had a slight improvement on the tapping score with a slight deterioration on the UHDRS score (top right quadrant), while another had a slightly improved UHDRS score with an annual decrease on the number of taps that was about average (bottom left quadrant). "+" = Improvement, "-" = Deterioration.

A common method for examining longitudinal changes in a variable is to plot it against time. This is shown in Figure 1 for two outcome variables: the first is a hand tapping score (a measure of psychomotor speed) which is the number of times a patient alternately taps two large buttons in 30 seconds with a single hand (Fig 1A), and the lower the score the worse the performance or disease state. The second is the UHDRS motor score, which is a clinical assessment based on a range of motor tasks, with higher values indicating worse performance or disease state (Fig 1B). Data for two individuals are shown (blue circles and orange squares) and it can be seen that there is a steady decline over time in the number of taps that these two patients were able to perform, with the orange patient decreasing at a faster rate (Fig 1A). UHDRS scores gradually increased (worsened) over time, but it is the blue patient that deteriorated faster on this measure (Fig 1B). There is also a good correlation between the two outcomes (Fig 1C), but this plot loses any reference to time and therefore the progression of the disease. Whilst both tapping scores and UHDRS are plotted in Figure 1C, this graph cannot distinguish between a patient that has gradually deteriorated on both measures, gradually improved on both measures, or initially deteriorated and then improved in response to a treatment – it is only the bivariate relationship that is displayed, which limits the usefulness of this graph for monitoring disease progression as this requires the variables to be graphed as a function of time. With scatterplots such as Figure 1A and 1B, it is easy to visualise how individuals change over time on each variable separately, but it is more difficult to observe the change in both tapping and UHDRS scores simultaneously. Furthermore, it would be difficult to examine many patients over time on one graph (or in separate panels) for these two variables in order to observe trends in the data, and this was the motivation for developing vector plots and path plots. These same two individuals are also highlighted (by colour) in Figures 2A, 3A and 3B so that it can be seen how this raw data can be represented by vectors.

The basic vector plot is shown in Figure 2A, where the base of each grey arrow (closed circles) are the initial UHDRS and tapping values, and the tips of the arrows are the final values; the length of the arrow therefore represents the amount of disease progression. Patients progress from the base of the arrow at the initial assessment to the tip of the arrow at the final assessment, with the net change being graphed. The patients in this particular study were not at the same stage of the disease upon entry into the study and therefore some of the variability in the initial scores represents patients at a different stage of the disease. It should be noted that these patients were followed up for different lengths of time (mean = 6.8 years, range = 5–8 years) and therefore the length of the vectors is not directly comparable in Figure 2 and Figure 3A, as one patient may have a longer arrow (i.e. greater apparent disease progression) simply because they have been followed up for a longer time. This is a shortcoming of the dataset and not the graphical method, and can be easily accommodated (see below). Several summary statistics can be added to the basic graph to assist in visualing general trends. The first is the mean vector (black arrow in Fig 2A), which is simply the average of the initial and final values for each variable. If the data are skewed or contain outliers, then the median vector could also be used as a more robust measure of central tendency. An alternate method of displaying the mean (or median) values is to plot the projection of the mean vector onto the *x *and *y *axes. This is shown in Figure 2B, where the blue circles on the *x *and *y *axes represent the mean initial values for the number of taps and UHDRS score, respectively. The red diamonds on the axes represent the mean final values, and the distance between them is the average change. This has the advantage of making it easier to estimate the mean values and has less clutter on the plotting region. Figure 2B also plots the bivariate interquartile range (IQR), which is interpreted in the same way as a univariate IQR: 50% of the initial (blue) and final (red) values lie within their respective ellipses. This gives a visual representation of the dispersion of the values as well as the overlap of the middle portion of the initial and final values. The shape of the ellipses also provides information on the correlation between the two variables at each time point; the ellipses would be circular with no correlation, and the more elongated the ellipses the greater the correlation.

Figure 2C uses two other techniques to highlight characteristics of the data. Instead of standard axes as in the previous graphs, the axes extend from the lowest to the highest values and thus indicate the range of the data [27]. In addition, the data at each time point are enclosed in convex polygons, making it easier to visually cluster the initial and final values. A convex hull is the smallest subset of points that when connected with line segments, enclose the entire set of points, and is conveniently graphed as a shaded polygon. This plot takes a macroscopic view of the data and highlights the range and any extreme scores. For this graph it can be seen that the initial values are within a narrower range than the final values for both the tapping and UHDRS scores, implying that patients are more alike at the initial observation and tend to diverge over time.

### Visualising rate of change or change scores

The previous graphs plotted the raw scores, which has the advantage of visualising the data in the units that they are measured. However, it will often be useful to adjust for initial differences, either because people may be at different stages of the disease upon entry into the study or due to natural heterogeneity in the sample. Adjusting for initial differences also makes it easier to compare changes between patients when they all start with a common baseline. This is shown in Figure 3A, where data for each variable are represented as change scores (final minus initial values). From this graph it can be seen that two patients clearly stand out; one improved on the tapping score and the other improved on the UHDRS score.

As mentioned above, these patients were followed up for different lengths of time and therefore the lengths of the vectors are not directly comparable. However the vectors can be normalised by dividing the change scores by the length of follow-up time for each patient, and this represents the rate of change (i.e. average change per year; Fig 3B). The relative lengths of the vectors has not changed much with this particular dataset, as the follow-up time for each subject, while not identical, was not vastly different.

**Figure 4:**
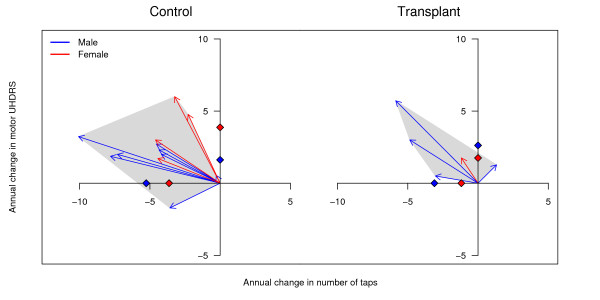
**Vector plots by sex and condition**. Vector plots of annual disease progression with sex encoded by different coloured arrows and data plotted separately for controls and individuals that had neurotransplantations. The grey polygons assist in visualising the population of values; the smaller the area of the polygon in the top left quadrant indicates less disease progression. The mean values for both sexes are shown on the axes, where it can be seen that male controls progressed faster on the tapping score, whereas females progressed faster on the UHDRS.

Further information can be added to these plots to examine differences between groups, such as between an experimental and control group, and these can be encoded by vectors of different colour, style, or thickness. In addition, the data can be stratified or conditioned on a real valued variable such as age, age of onset, or CAG repeat length, where subsets of the data are plotted in different panels for different levels of the conditioning variable [11]. For example, in Figure 4, the data are plotted separately for controls and patients that received neural transplants. The mean values for both sexes are shown on the axes, where it can be seen that in the control patients, males progressed faster on the tapping score whereas females progressed faster on the UHDRS score (there was only one female transplant patient). It can also be seen that the transplant patients deteriorated at a slower rate than the controls, especially on the tapping score. There are only five females in this dataset (and a total sample size of 17), which is too low for any strong inferences to be drawn, but highlights the type of patterns that can be detected. The grey polygons aid in visualising the population of values; the smaller the area of the polygon in the top left quadrant, the less disease progression. The polygons also highlight the extreme scores, and so their area may be influenced by one or two outliers, but they nevertheless provide an impression of how fast patients in the two groups are progressing. Alternatively, bivariate IQRs could be plotted (either for all the patients in a panel or separately for each sex), which would provide summary information on the middle portion of these change scores rather than those with the largest change. It is often of interest to examine the relationship between baseline values and the rate of disease progression [28], and this can be easily accommodated by using different types of vectors; for example, vectors of different colour for patients with high versus low baseline values.

**Figure 5:**
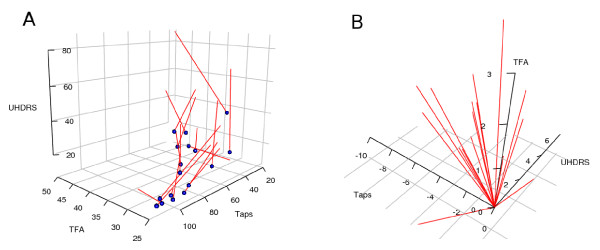
**Vector plots of three outcome variables**. Raw values for the UHDRS motor score, number of taps in 30 seconds, and Total Functional Assessment (TFA) score are plotted (A). The blue spheres are the initial values, and the tips of the red lines are the final values (arrowheads are omitted). Generally, the initial values are in the lower front corner and the vectors progress towards the top back corner. The normalised change scores are shown in (B) and the graph is oriented to show the general trend and to highlight the two patients that differ from the rest. The ability to rotate the graphs in real time is necessary to fully appreciate the distribution and directions of the vectors in 3D.

One drawback of vector plots as used in Figures 2, 3, 4 and 5 is that only the initial and final values are graphed, and other measurements that may have been taken at intermediate time points are ignored, and thus all the available data are not used. However, it possible to use all the data to estimate the initial and final values by fitting a regression line through the data (e.g. by least squares or a robust method) and use the predicted values at the initial and final assessments rather than the actual values (similar to the method used to obtain the values for the path plots, which is discussed below). This has the virtue of using all of the observations on each patient to construct the vector, and it can be useful if the outcome variables fluctuate from one observation to the next. However, the interpretation of a change from an initial assessment to a final assessment is more intuitive (which is why these values were used in the present paper), and may be all that is available in a pre versus post design with only two time points.

### Adding a third variable

A third axis can be added to include another outcome variable, allowing six pieces of information per patient to be graphed. An example is shown in Figure 5, where values on the Total Functional Assessment (TFA) score – another clinical measure – are included on a third axis. This requires 3-dimensional graphs, and the ability to rotate the graph in real time is necessary to fully appreciate the orientation of the vectors. The graphs in Figure 5 were created with the rgl package [29], which enables zooming and rotation in any direction. In addition, making the vector plots interactive by integrating them into multivariate visualisation tools such as GGobi [13] would enable techniques such as 'brushing' to be used. Brushing involves using the cursor to select a graphical object in one plot, such as a vector, and data corresponding to the same individual (or a class that the individual is in) are highlighted in other plots. For example, putting the cursor over the only vector in the top right quadrant of Figure 3B would highlight this individual in another graph, which might plot age of onset for each sex separately as a dot plot. One could then check if this patient had a particularly early or late age of onset and their sex. Values for individual patients can be linked up across multiple panels and variables, which provides a powerful method to examine the multivariate nature of data obtained in many clinical studies.

### Tracking disease over time

**Figure 6:**
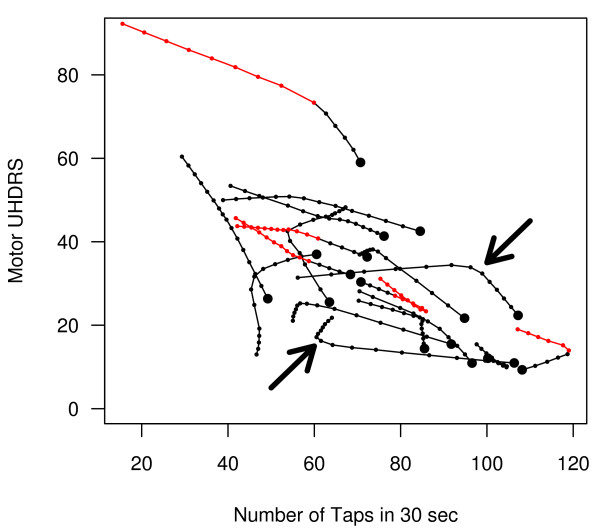
**Path plot**. This graph shows the path of disease progression from the first (large black circles) to the final (end of the line) observation. The small dots along the line are at six month intervals and therefore represent time. The first assessment after patients received a neural transplantation are shown in red (black-only paths are controls) and any changes in disease trajectory post-transplant can be easily visualised. Different patterns of progression can also be seen; for example, some patients have an abrupt stop in the deterioration of the tapping score after a transplant (lower arrow), while others progressively perform fewer taps with a relatively stable UHDRS score (higher arrow).

The previous 2D and 3D graphs simply examined what the patients were like at an initial and final time point and ignored the path by which patients arrived at their final disease state. Did some progress at a steady rate, while others progressed slowly initially and then accelerated as the disease progressed, while others had a fast initial deterioration which then levelled off? These questions can be addressed by plotting the data as shown in Figure 6. To generate this plot, a locally estimated regression (loess) was used to fit a smooth regression line through the data in Figure 1A and 1B (see Additional Files 4 and 5). The predicted values for the number of taps and UHDRS scores at each time point are then plotted against each other, and this line represents a *path *in Wilkinson's system of describing graphical components [30]. The smoothness of the line can be adjusted to follow the data closely, which allows smaller trends and fluctuations to be detected, but will also pick up 'uninteresting' changes such as the natural variability in the repeated observations and measurement error. Alternatively, a stronger smoothing function can be used to average over the smaller fluctuations and observe only the larger trends in the data. Figure 6 used strong smoothing to highlight the overall trends, and the large black circles in this figure are the initial values, and the end of the paths (lines) are the final values (arrow heads are omitted to avoid clutter). The small dots along the line serve as a time stamp and are six months apart (the intervals at which the data were collected); this allows one to not only observe disease trajectories but how the trajectories evolve over time. Equally spaced dots (alternatively, equal lengths of line segments between dots) indicates a consistent rate of disease progression. Dots that are close together imply little progression over time whereas dots that are farther apart indicate faster progression (or improvement). With this particular dataset, some patients received neural transplantations of human foetal striatal tissue into the striatum. For the transplant patients, the line changes from black to red at the first post transplant assessment, allowing any changes in disease trajectories to be visualised after treatment. More generally, this type of plot is suitable to visualise the effect of any intervention where multiple baseline and post-treatment observations are recorded. In addition, it is a method of distinguishing subgroups of patients that progress differently over time (e.g. steady rate, accelerating, levelling-off), and which may be related to other environmental or biological factors. For example, there is increasing evidence for heterogeneity in Parkinson's disease [31-33], with faster progression in a subgroup of patients that are older, have a non-tremor dominant phenotype, and deficits in semantic fluency [34]. A path plot might be useful to visually classify individuals based on their disease progression, and one could then examine whether the subgroups differ in other respects such as gene expression, imaging results, or known risk factors for the disease. Path plots can also include a third variable but are difficult to visualise in print, and so an animated GIF can be found in Additional File 6, which plots TFA scores on the third axis.

### Advantages of vector and path plots

There are a number of advantages of using these plots for exploratory data analysis. The first, which was already mentioned in the introduction, is that they can facilitate the detection of multivariate outliers, and understanding the structure of the data will assist in the final modelling and analysis. Second, information on individual patients can be graphed, allowing comparisons of individuals with group trends; in clinical studies it is often important to observe data at the level of the individual patient rather than simply averaged responses. Third, summary statistics such as mean vectors and bivariate interquartile ranges can be included on the graph, along with visual guides such as convex polygons to describe the population of vectors. Fourth, since the length of the (normalised) vector is on a ratio scale, its interpretation is straightforward: a person with a vector twice as long as another has progressed twice as fast. Finally, missing values or a different number of observations between patients do not pose any particular difficulty for these plots. This is an important attribute because it is not uncommon to have missing data in longitudinal studies – for example if patients are unavailable for a particular assessment.

### Disadvantages

The main disadvantage is that these plots are not readily available in major statistical or graphical software packages. Some of the R code is provided, but familiarity with the R language is required in order to use it. It is hoped that these methods will become more widely available in the future.

### Further extensions

Vector plots were used in the present paper to compare changes on a clinical measure of motor dysfunction and a simple hand tapping measure that is under evaluation as a potential biomarker in HD. Other potential biomarkers such as quantitative oculometry [35] and olfactory functioning [36] can discriminate between HD patients and controls in cross-sectional studies, and whole-brain atrophy has been used with a six month follow-up period [37]. If longitudinal data were available for these methods, vector and path plots would be useful for determining which biomarker tracks disease progression better. These plots are also suitable for comparing two different assessment methods such as a novel method with a gold-standard, in addition to other existing graphical methods [38-41], and assuming that one has a gold-standard by which to compare these novel methods or potential biomarkers. Another use for these plots is to examine left versus right asymmetries. For example, Parkinson's disease often presents with one side more affected than the other (unilateral onset is a UK Parkinson's Disease Society Brain Bank criteria for diagnosis [42]) and changes in asymmetry could be tracked over time. The data used in this paper were from a longitudinal study with multiple observations, but vector plots can also be used for simple pre versus post designs. In addition, there is nothing restricting such plots to human clinical data and they would also be suitable for many preclinical animal studies.

Instead of plotting the actual values of the outcome variables, the parameters of summary or distributional statistics could also be graphed. For example, in addition to the longitudinal study in the original paper [15], it also contained a cross-sectional study comparing tapping scores between HD patients and controls. In the cross-sectional study, not only were the number of taps determined, but also the variability in the time between successive taps (the inter-tap interval). If this data was also collected for the longitudinal study, then both the number of taps and the variability of the inter-tap interval could have been plotted over time. In other words, the parameters (mean and variance) of a distribution of taps could have been plotted for each subject at different time points (in practice, the interdecile range was used rather than the variance as the distributions had some outliers). While plotting parameter values for distributions is more abstract then plotting raw data values, these plots can be used to visualise changes in parameter space over time and they also have a straightforward interpretation.

The thickness of the vectors could also be used to encode information such as class membership (e.g. male vs. female), in which case only two levels of thickness would be used. Alternatively, the vector thickness could be used to represent a continuous variable such as the variability in the original measurements, which would allow for different patterns of variability to be visualised.

It might be difficult to observe individual values and their trajectories if there are many patients. This can be partly overcome by using semi-transparent vectors (also referred to as *alpha blending *or *splatting*) so that vectors that are underneath others can be partially seen. Alternatively, subsets of patients could be selected and plotted rather than all the patients at once. Subsetting can be achieved by breaking the data down by groups or conditions, or random subsets of the data can be plotted in a number of different panels so that all the data can be seen at once.

## Conclusion

Vector plots – using either raw data or change-scores – and path plots provide novel graphical techniques for visualising how individual patients or subjects change over time on multiple variables. These plots are useful for comparing groups on two or more variables, detecting multivariate outliers, and detecting subgroups of patients that have different disease trajectories. They are a useful addition to standard graphical exploratory data analysis methods and can be used to gain new insights into longitudinal data and thus the natural progression of many conditions, as well as how treatments affect disease trajectories.

## Competing interests

The authors declare that they have no competing interests.

## Authors' contributions

SEL planned the present study, carried out the analysis, and wrote the paper. AWM helped plan the present study, planned the original study, collected the patient data, and helped write the paper. SLM helped collect the data, and RAB planned the original study, helped collect the patient data, and helped write the paper. All authors read and approved the final manuscript.

## Pre-publication history

The pre-publication history for this paper can be accessed here:

http://www.biomedcentral.com/1471-2288/9/32/prepub

## Supplementary Material

Additional file 1 **Initial and final data**. The hand-tapping, UHDRS and TFA data are provided. Variables starting with 't0' are initial values, and those starting with 'tf' are final values. The variable 'years' is the length of follow-up time. Click here for file

Additional file 2 **Longitudinal data**. Longitudinal hand-tapping and UHDRS data are provided. Click here for file

Additional file 3 **R code**. R functions to produce graphs similar to those in the figures are provided. Click here for file

Additional file 4 **Individual plots of changes in hand tapping over time**. Hand tapping data are plotted over time for each patient separately along with a loess regression line. Data are organised in order of increasing median number of taps from left to right, bottom to top. Click here for file

Additional file 5 **Individual plots of changes in UHDRS over time**. UHDRS data are plotted over time for each patient separately along with a loess regression line. Data are organised in order of increasing median UHDRS score from left to right, bottom to top. Click here for file

Additional file 6 **Animated 3D path plot**. The path of disease progression from the first (blue spheres) to the final observation for number of taps, UHDRS, and TFA scores, and can be viewed with a web browser. Click here for file
