# Supplementary figures and images for: Visualising disease progression on multiple variables with vector plots and path plots
Source: BMC Med Res Methodol. 2009 May 27;9:32. doi: 10.1186/1471-2288-9-32 (PMC2693505; doi:10.1186/1471-2288-9-32)

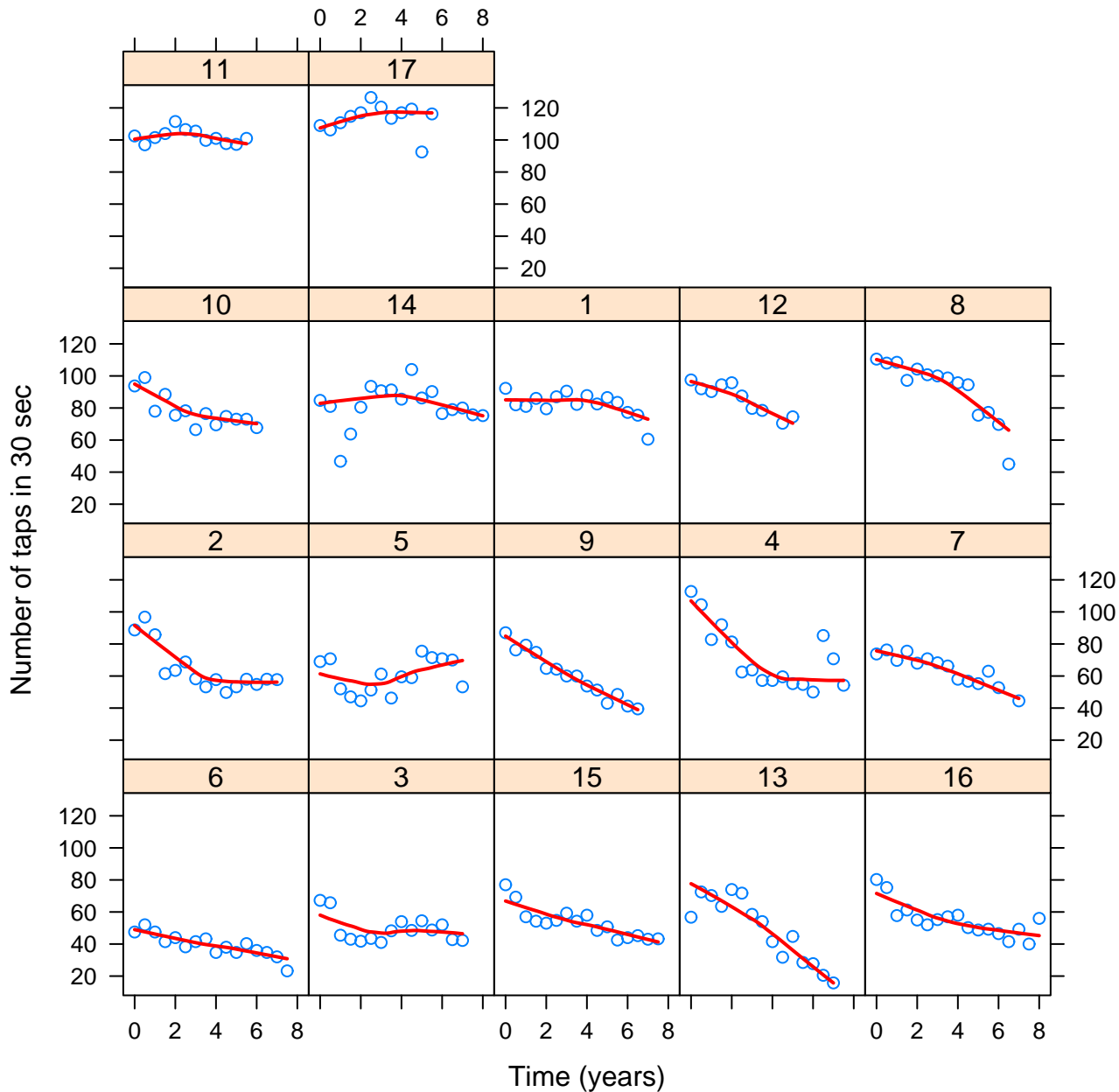

Supplement: Additional file 4 — Individual plots of changes in hand tapping over time. Hand tapping data are plotted over time for each patient separately along with a loess regression line. Data are organised in order of increasing median number of taps from left to right, bottom to top. [file 1471-2288-9-32-S4.pdf]

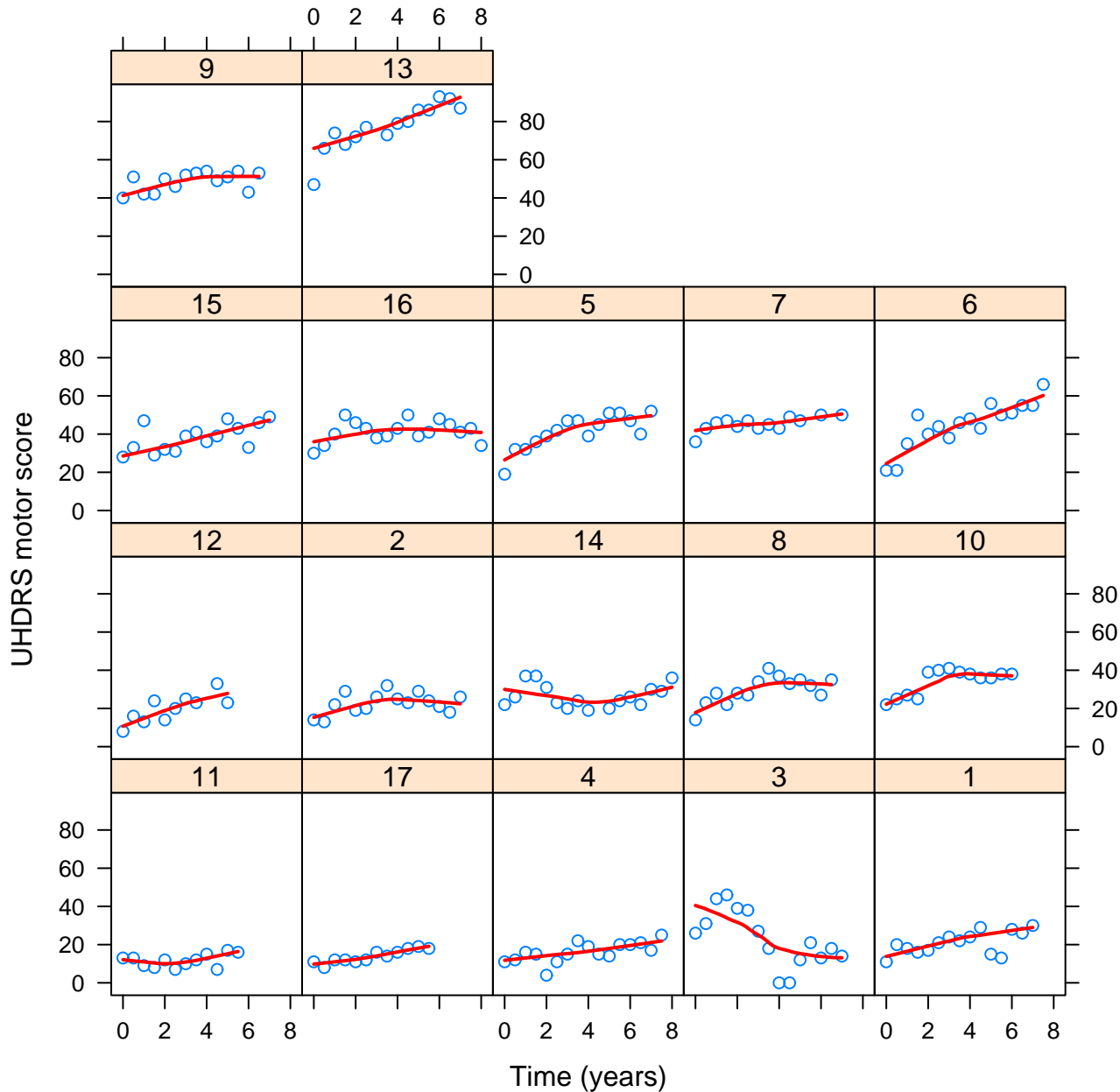

Supplement: Additional file 5 — Individual plots of changes in UHDRS over time. UHDRS data are plotted over time for each patient separately along with a loess regression line. Data are organised in order of increasing median UHDRS score from left to right, bottom to top. [file 1471-2288-9-32-S5.pdf]

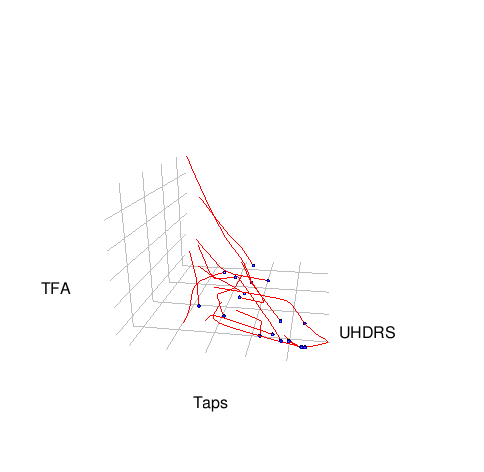

Supplement: Additional file 6 — Animated 3D path plot. The path of disease progression from the first (blue spheres) to the final observation for number of taps, UHDRS, and TFA scores, and can be viewed with a web browser. [file 1471-2288-9-32-S6.zip › Add6.gif]
